# Differences in COVID-19 Hospitalizations by Self-Reported Race and Ethnicity in a Hospital in Honolulu, Hawaii

**DOI:** 10.5888/pcd19.220114

**Published:** 2022-11-17

**Authors:** Brendan K. Seto, Laura Nishizaki, Gerard Akaka, Jo Ann Kimura, Todd B. Seto

**Affiliations:** 1 John A. Burns School of Medicine, University of Hawaii, Honolulu, Hawaii; 2 The Queen’s Medical Center, Honolulu, Hawaii

## Abstract

**Introduction:**

The true extent of racial and ethnic disparities in COVID-19 hospitalizations may be hidden by misclassification of race and ethnicity. This study aimed to quantify this inaccuracy in a hospital’s electronic medical record (EMR) against the gold standard of self-identification and then project data onto state-level COVID-19 hospitalizations by self-identified race and ethnicity.

**Methods:**

To identify misclassification of race and ethnicity in the EMRs of a hospital in Honolulu, Hawaii, research and quality improvement staff members surveyed all available patients (N = 847) in 5 cohorts in 2007, 2008, 2010, 2013, and 2020 at randomly selected hospital and ambulatory units. The survey asked patients to self-identify up to 12 races and ethnicities. We compared these data with data from EMRs. We then estimated the number of COVID-19 hospitalizations by projecting racial misclassifications onto publicly available data. We determined significant differences via simulation-constructed medians and 95% CIs.

**Results:**

EMR–based and self-identified race and ethnicity were the same in 86.5% of the sample. Native Hawaiians (79.2%) were significantly less likely than non–Native Hawaiians (89.4%) to be correctly classified on initial analysis; this difference was driven by Native Hawaiians being more likely than non–Native Hawaiians to be multiracial (93.4% vs 30.3%). When restricted to multiracial patients only, we found no significant difference in accuracy (*P* = .32). The number of COVID-19–related hospitalizations was 8.7% higher among Native Hawaiians and 3.9% higher among Pacific Islanders when we projected self-identified race and ethnicity rather than using EMR data.

**Conclusion:**

Using self-identified rather than hospital EMR data on race and ethnicity may uncover further disparities in COVID-19 hospitalizations.

**Summary**

**What is already known on this topic?**

Racial and ethnic disparities in the number of COVID-19 hospitalizations exist, and data on race and ethnicity in hospital electronic medical records are known to be inaccurate for non-White populations.

**What is added by this report?**

We described the inaccuracy of race and ethnicity identification in a large multiracial population, then projected these findings onto publicly available COVID-19 hospitalization data to estimate disparities by self-identified, rather than hospital electronic medical record–based, race and ethnicity.

**What are the implications for public health practice?**

Accurate race and ethnicity data are essential for reliably measuring disparities. Race and ethnicity data, especially in multiracial populations, should be confirmed when possible, and reporting practices could be evaluated to promote reliable results.

## Introduction

Despite efforts to address health inequities, there are persistent — and sometimes substantial — disparities in health among some racial and ethnic groups in the US and worldwide (1). Efforts to understand the magnitude and causes of these disparities are often complicated by the lack of consensus on how one’s race and ethnicity are defined. Although several approaches exist (2–4), the most common method and the current gold standard for determining race and ethnicity is self-identification, with federal best practices from the Centers for Medicare and Medicaid Services (CMS) available to guide standardized data collection, including information to address key challenges in collecting these data (5).

However, the accuracy of data on race and ethnicity in large administrative data sets may be lacking, especially in non-White patient populations; the accuracy of such data is estimated to be 88% among the US patient population overall and 66% in non-White patient populations (6–17). Accuracy may be even less reliable among the increasing number of people who identify as multiracial, which is especially common among young people. To our knowledge, only 2 studies have investigated the accuracy of racial and ethnic information of multiracial populations in hospitals (6,13). Both studies showed less accuracy in correctly identifying race and ethnicity among multiracial patients (21% accuracy) than among nonmultiracial patients (65% accuracy) in the electronic medical record (EMR), although the number of multiracial patients in both studies was small (0.4% and 4.3% of the patient population). US Census data show that people younger than 18 years are nearly twice as likely as people aged 18 years or older to identify as multiracial (15.1% vs 8.8%) and that the number of people identifying as multiracial increased by 276% from 2010 to 2020 (18). Thus, the challenges of identifying a person’s race and ethnicity will continue to grow. It is important to explore the implications of these challenges by studying their potential impact in a highly diverse, multiracial, majority–minority population.

COVID-19 has presented a challenge to our modern lives, but many racial and ethnic minority groups have a disproportionate burden of cases, hospitalizations, long-term complications, and deaths (19,20). As of March 2021, Native Hawaiians and Pacific Islanders had the highest death rate of any racial or ethnic group in 18 of the 20 states that reported deaths among those 2 groups (21). In Hawaii, Pacific Islanders account for 5% of the population but 22% of COVID-19 cases and deaths (22). A fundamental requirement for understanding the magnitude and causes of these disparities is accurate data on race and ethnicity. For example, publicly available data from the Hawaii Department of Health show that Native Hawaiians and Pacific Islanders make up a disproportionate number of COVID-19 hospitalizations. But this number may have been underreported — and the true disparities underestimated — if the race and ethnicity of some patients have been misclassified.

The objective of our study was to determine the accuracy of race and ethnicity data in a hospital EMR system compared with self-identified data and then use this information to determine how the magnitude of COVID-19 disparities among racial and ethnic groups would change if patients were correctly classified.

## Methods

### Accuracy of EMR-based data on race and ethnicity vs self-identification

The study population consisted of patients at The Queen’s Medical Center (QMC), a 500-bed university-affiliated tertiary care hospital in Honolulu, Hawaii. QMC is the largest health care system in the state and serves as the primary referral center for the Pacific Basin.

We obtained survey data from QMC. Both inpatients and outpatients were recruited to participate in a survey administered by trained data collectors who visited randomly selected hospital and ambulatory units and asked all available patients if they would participate. Data were collected in 5 cohorts as part of an ongoing quality assurance project conducted by hospital staff during 5 years from 2007 through 2020 (2007, 2008, 2010, 2013, 2020). The major inclusion criterion was QMC patients who were provided care on the day of data collection. Non–English-speaking patients were included if a friend or family member was able to interpret. We excluded patients who were in intensive care units, unable to respond verbally, declined participation, or lacked an accessible EMR at QMC at the time of data collection. The QMC institutional review board approved the study protocol.

Patients were first asked to list all their races and ethnicities. Twelve spaces were provided for entries, but no patient listed more than 10 races or ethnicities. They were then asked to select the one that they identified with the most; this was defined as the self-identified race and ethnicity. Patient responses were aggregated to modified 1997 OMB minimal reporting guidelines that disaggregated Native Hawaiians and Pacific Islanders into 2 groups to mirror COVID-19 reporting practices in the state. OMB guidelines encourage this additional granularity when possible and relevant to the population (3). A separate multiracial indicator was created to identify participants who reported at least 2 racial and ethnic categories per 1997 OMB guidelines (3).

The process used at QMC to identify patient race and ethnicity for the EMR was developed in 2010 as part of a statewide collaborative among all acute care hospitals in Hawaii. The process was based on a framework developed by the Health Research & Educational Trust and implemented statewide via standardized tools and training (23). All patients were asked to identify the one race or ethnicity that they identified with the most. For patients who selected multiple races and ethnicities, hospital staff members were instructed to follow a hierarchical algorithm used by the Hawaii Department of Health (23). By hospital policy, once a patient identified a race and ethnicity, the hospital did not ask the question again during future visits; thus, this framework applies only to first-time entries since 2010 (23).

Survey responses on race and ethnicity for each patient were then compared with the race and ethnicity noted in their EMR (Epic Systems Corporation), the gold-standard for self-identification. Patients provided their hospital medical record number in their survey response, which study team members used to link surveys to the EMR. We defined accuracy as the sensitivity of the EMR in predicting a patient’s self-identification. EMR–based data were considered accurate if they matched the self-identification and inaccurate if they did not. We calculated accuracy as the total number of hospital EMR entries that matched self-identification divided by the total number of surveys. We also calculated positive predictive values for each self-identification and determined significance for both measures via analysis of variance followed by pairwise Welch *t* tests. Reasons for lack of agreement were grouped into 3 categories: 1) the race and ethnicity listed in the EMR differed from self-identification but was listed in the patient’s original list of self-reported races and ethnicities, 2) the race and ethnicity listed in the EMR was not included in the patient’s original list of self-reported races and ethnicities, and 3) no entry for race was found in the EMR. When we found no entry for race (n = 6), we categorized patients as belonging in the “Other” group and set their default status as “inaccurate EMR entry.” We generated a confusion matrix (a table used to define the performance of a classification algorithm) to explore patterns in disagreement by race and ethnicity.

We conducted additional analyses to compare accuracy among subpopulations, such as multiracial versus single-racial, patients with different self-identifications, and patients in different cohorts. Subsequent analyses consisted of χ^2^ tests, paired Welch *t* tests, or analysis of variance followed by paired Welch *t* tests, as appropriate.

### Impact on statewide COVID-19 racial and ethnic health disparities

Our data on the number of COVID-19–related hospitalizations were publicly available from the Hawaii Department of Health, current as of January 12, 2022, when the state stopped publicly reporting the number of hospitalizations by race and ethnicity. During the COVID-19 pandemic, hospitals and laboratories were required to submit detailed data on hospitalizations, including data on race and ethnicity, age, and other demographic characteristics of patients. Thus, statewide, publicly reported data on race and ethnicity data were derived directly from hospital EMR data. All data are available at https://health.hawaii.gov/coronavirusdisease2019/current-situation-in-hawaii (24). QMC accounted for 45% of all COVID-19 hospitalizations in the state, with patient demographics closely matching the characteristics of all COVID-19–hospitalized patients statewide.

We explored projected COVID-19 hospitalization rates by self-identification vs EMR-based data by using a simulation (25) in 3 steps. In the first step, we created a pseudo-population matrix based on publicly available COVID-19 hospitalization data from the Hawaii Department of Health. This pseudo-population matrix had a row for each hospitalization and a column containing a hospital-reported race and ethnicity in proportion to the state’s racial breakdown. Second, each entry (a patient’s EMR-based race and ethnicity) was then randomly assigned a projected self-identified race and ethnicity, with probabilities based on the race and ethnicity confusion matrix derived from the QMC surveys. This value was added as a second column. In the third step, we tallied total self-identified race and ethnicity estimates for the projected population. Steps 2 and 3 were repeated 1,000 times to generate a distribution, from which a median and 95% CIs for each self-identified race and ethnicity were derived. An initial χ^2^ test was conducted to determine whether the median projected distribution of self-identification differed significantly from the state’s estimates based on hospital EMR data. Projected self-identified population proportions by race and ethnicity were considered significantly different from the proportions among the hospital-derived race and ethnicity if the hospital-derived number fell below the 2.5th or above the 97.5th percentile (ie, outside a 95% CI); *P* values were determined by assuming simulation results were normally distributed. We created a density plot to compare the distribution of self-identified race and ethnicity with the distribution of EMR–based race and ethnicity for each racial and ethnic group.

All analyses were conducted by using R statistical software for Mac, version 4.0.5 (R Foundation for Statistical Computing). We also used tidyverse and Mosaic for data manipulation and general utility (26,27). 

## Results

### Accuracy of EMR-based race and ethnicity vs self-identification

A total of 847 surveys were obtained from QMC. Participants were evenly distributed among the 5 cohorts, with each cohort consisting of more than 100 responses.

Our study population was majority–minority, with no single self-identified race and ethnicity reported by more than 50% of the survey participants. The largest self-identified groups reported by survey were Native Hawaiian (21.7%) and Pacific Islander (18.1%), and Asian (33.3%), followed by non-Hispanic White (21.2%), Hispanic (2.7%), non-Hispanic Black (1.9%), and Other (1.1%). This distribution closely matched the distribution of EMR–based data, which was the following: Native Hawaiian (18.8%) and Pacific Islander (17.2%), and Asian (33.0%), followed by non-Hispanic White (22.5%), Hispanic (2.5%), non-Hispanic Black (2.2%), and unknown or missing (3.8%). Our survey sample was similar to the statewide population of people reported to have been hospitalized with COVID-19, but the sample had more Native Hawaiians and Pacific Islanders relative to the state’s general population and fewer Asians and non-Hispanic White people.

Forty-four percent (373 of 847) of survey participants listed more than 1 race and ethnicity. Among these multiracial participants, the average number of races and ethnicities reported was 3.1, with a maximum of 10. Participants who self-identified as Native Hawaiian were more likely than all other groups to be multiracial (92.4%), while Pacific Islanders were the least likely to be multiracial (15.0%). Approximately one-quarter (26.3%) of self-identified Asian participants, 43.6% of self-identified non-Hispanic White participants (Table 1), 47.8% of self-identified Hispanic participants, and 43.8% of self-identified non-Hispanic Black participants identified as multiracial. Self-identified Native Hawaiians who were multiracial also listed significantly more races and ethnicities than other groups (Native Hawaiian, 3.3; non–Native Hawaiian, 2.8; *t* = −3.39; *P* < .001).

**Table 1:** Demographic Characteristics of Patient Respondents to a Hospital Race Validation Survey,^a^ Patients in a Hospital Electronic Medical Record System,^b^ and Patients Included in State-Reported COVID-19–Related Hospitalizations^c^

Item	Asian	Native Hawaiian	Pacific Islander	Non-Hispanic White
**Patient survey population**				
Overall, %	33.3	21.7	18.1	21.2
Multiracial, %	26.3	92.4	15.0	43.6
No. of races listed, mean (SD)	2.8 (0.9)	3.3 (1.5)	3.0 (1.1)	2.5 (1.2)
Age, mean (SD), y	53.2 (19.3)	50.5 (13.5)	49.1 (17.9)	56.1 (16.3)
**Hospital electronic medical records,^b^ %**	33.0	18.8	17.2	22.5
**State-reported COVID-19–related hospitalizations,^c^ %**	37.8	22.8	17.8	15.4

a Survey data were collected in 5 cohorts as part of an ongoing quality assurance project conducted by The Queen’s Medical Center, Honolulu, Hawaii, during 5 years from 2007 through 2020 (2007, 2008, 2010, 2013, 2020).

b Extracted from The Queen’s Medical Center’s electronic medical records.

c Extracted from publicly available data on 4,041 COVID-19–related hospitalizations from the Hawaii Department of Health, current as of January 12, 2022 (24).

The overall agreement between self-identified race and ethnicity and EMR-based race and ethnicity was 86.5% (733 accurate, 114 inaccurate). Of the nonagreements, 43 (37.7%) of the EMR-based races and ethnicities matched their self-reported options, but it was not the race and ethnicity the patient identified with the most; 65 (57.0%) were complete mismatches, where the EMR-based race and ethnicity were not listed by the patient at all; and 6 (5.3%) were the result of the EMR lacking any entry for race (Table 2).

**Table 2:** Confusion Matrix Showing Agreement Between Hospital Electronic Medical Records and Self-Identified Race and Ethnicity Among 847 Patients in a Hospital in Honolulu, Hawaii^a^

Self-identified race and ethnicity	Hospital EMR-based race and ethnicity						
	Asian	Non-Hispanic Black	Non-Hispanic White	Native Hawaiian	Pacific Islander	Other	Positive predictive value
Asian	259	0	8	5	0	9	0.92
Non-Hispanic Black	0	16	0	0	0	0	1.00
Non-Hispanic White	3	0	162	6	1	7	0.91
Native Hawaiian	13	2	12	145	3	8	0.79
Pacific Islander	2	0	1	0	139	11	0.91
Other	2	1	7	3	2	18	0.55
Sensitivity^b^	.93	.84	.85	.91	.96	.34	—

a Survey data were collected in 5 cohorts as part of an ongoing quality assurance project conducted by The Queen’s Medical Center, Honolulu, Hawaii, during 5 years from 2007 through 2020 (2007, 2008, 2010, 2013, 2020).

b Overall accuracy of data (sensitivity) = 0.88.

**Figure 1:**
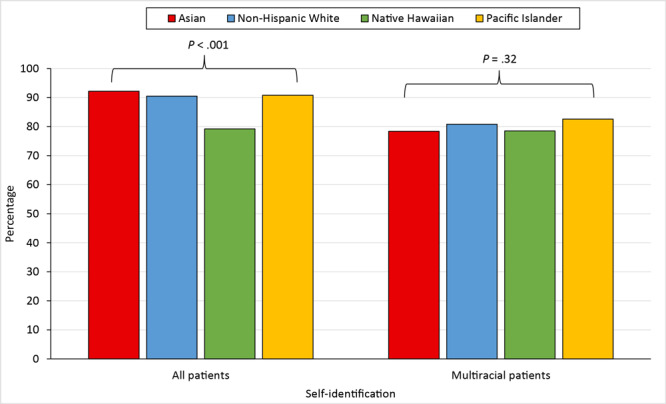
Overall accuracy of race and ethnicity in the electronic medical records of patients in a hospital in Honolulu, Hawaii. Overall accuracy was defined as the total number of hospital electronic medical record entries that matched the self-identified description divided by the total number of surveys.

The accuracy of EMR-based race and ethnicity was significantly lower for Native Hawaiian patients (79.2%) than for Asian (92.2%), non-Hispanic White (90.5%), and Pacific Islander patients (90.8%) (Figure 1). (Non-Hispanic Black and Hispanic patient populations were considered too small for reliable and comparable analysis.) This disparity was driven largely by Native Hawaiians being more likely to be multiracial, as patients who were multiracial were significantly less likely to be categorized correctly in the EMR (78.0% vs 94.5%; *P* < .001). When we restricted our analysis to multiracial patients only, we found no significant differences (*P* = .32) in accuracy among Native Hawaiian (78.5%), Asian (78.4%), non-Hispanic White (80.8%), and Pacific Islander patients (82.6%) (Figure 1). We found no significant difference in accuracy among the 162 patients who reported 2 races and/or ethnicities and the 210 patients who reported more than 2 (80.9% vs 75.7%, *P* = .23).

**Table:** 

Self-identification	All patients, %	*P* value	Multiracial patients, %	*P* value
Asian	92.2	<.001	78.4	.32
Non-Hispanic White	90.5		80.8	
Native Hawaiian	79.2		78.5	
Pacific Islander	90.8		82.6	

Accuracy varied by year with no apparent trend (2007, 82.4%; 2008, 87.5%; 2010, 92.1%; 2013, 90.2%; 2020, 85.6%). We found no differences in accuracy by patient age (*t *= 1.59; *P* = .11).

### Impact on statewide COVID-19 racial and ethnic health disparities

As of January 12, 2022, Hawaii had 4,041 COVID-19–related hospitalizations. Asian patients accounted for the largest percentage of COVID-19–related hospitalizations (37.8%), followed by Native Hawaiian (22.8%), Pacific Islander (17.8%), non-Hispanic White (15.4%), and patients of other races and ethnicities (6.2%). Native Hawaiians and Pacific Islanders were overrepresented relative to their share of the state’s population, whereas Asian and non-Hispanic White patients were underrepresented. However, the racial and ethnic distribution of COVID-19–related hospitalizations in Hawaii was similar to the distribution in our EMR data, indicating that the demographic characteristics of patients with COVID-19–related hospitalizations were similar to the demographic characteristics of patients who were hospitalized before COVID-19.

**Figure 2:**
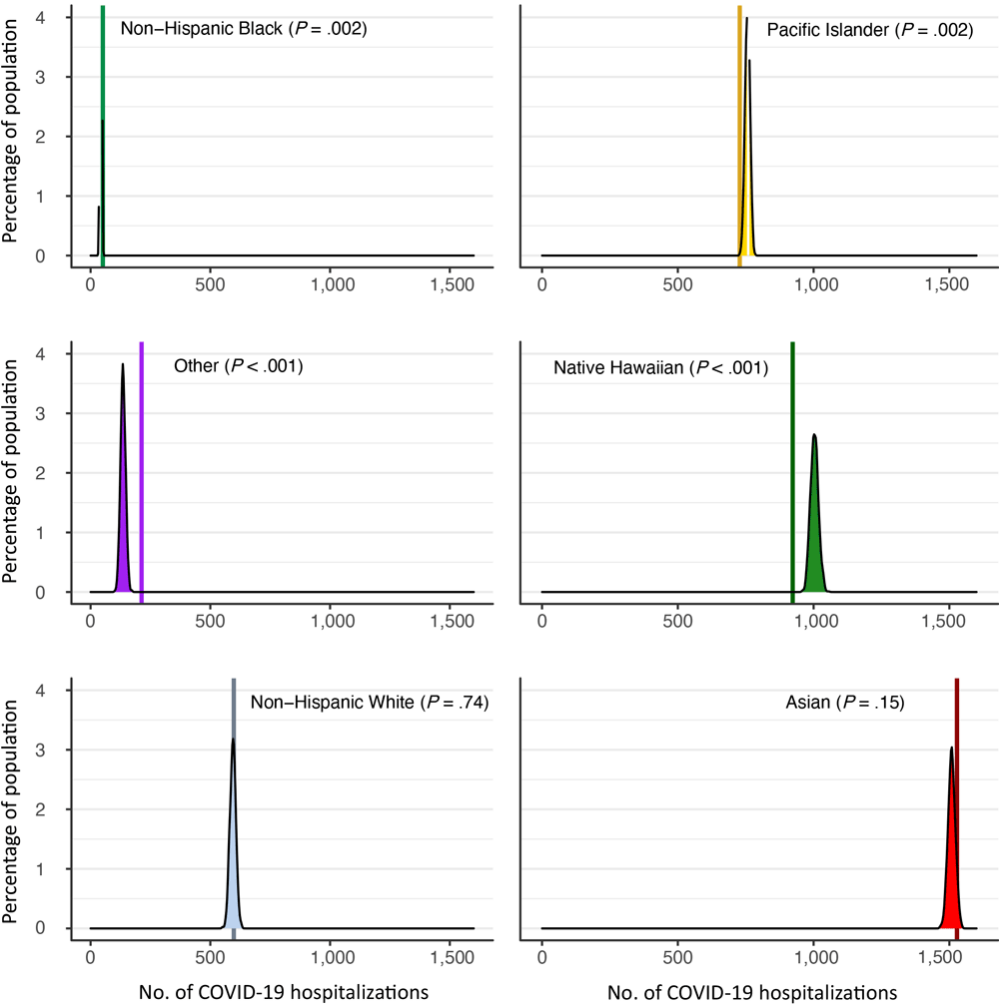
Results for a simulation of COVID-19–related hospitalizations that compared the distribution of adjusted self-identified race and ethnicity (simulated distribution) with the distribution of state-reported race and ethnicity (solid vertical lines). “Other” refers to any patient whose self-reported race did not match predefined categories (eg, “metropolitan,” “mixed,” blank response).

The overall differences between the adjusted and original COVID-19–related hospitalizations were significant (χ^2^ = 22.0, *P* < .001). The simulated distributions showed that projected COVID-19–related hospitalizations among self-identified Native Hawaiians and Pacific Islanders were significantly higher than state estimates, whereas projected COVID-19–related hospitalizations among the “other” races were significantly lower (Figure 2). The median number of COVID-19–related hospitalizations among Native Hawaiian patients was 8.7% higher (1,003 vs 923 hospitalizations) when self-identification rather than EMR-based data were used, and the overall increase in population share was 2.0 percentage points (from 22.8% of the population to 24.8%). The number of COVID-19–related hospitalizations also was higher when self-identification was used among Pacific Islander patients in both total numbers (+3.8%, from 728 hospitalizations to 756) and population share (+0.7 percentage points, from 18.0% to 18.7%); we found lower median numbers of COVID-19–related hospitalizations among all other races and ethnicities when we used self-identification rather than EMR-based data (Figure 2). While most of the newly identified Pacific Islander patients were reclassified from the EMR-based “other” race category, patients newly identified as Native Hawaiian came from many different EMR-based categories (Table 2).

**Table:** 

Race and ethnicity	State-reported no. of hospitalizations	Adjusted no. of hospitalizations	Percentage change	*P* value
Asian	1,528	1,509	Minus 1.3%	.15
Non-Hispanic White	598	594	Minus 0.6%	.74
Native Hawaiian	923	1,003	Plus 8.7%	<.001
Pacific Islander	728	756	Plus 3.4%	.002
Non-Hispanic Black	51	43	Minus 15.8%	.002
Other	213	135	Minus 36.4%	<.001

## Discussion

The accuracy of race and ethnicity in the EMR system of our study hospital, which has a diverse and multiracial population, was similar to the accuracy of the gold-standard of self-identification (86.5% accuracy for both). Yet even the slight disagreement in categorization was enough to affect health disparities in COVID-19–related hospitalization rates in Hawaii: the median number of COVID-19–related hospitalizations was 8.7% higher among Native Hawaiians and 3.8% higher among Pacific Islanders when we used self-identified data on race and ethnicity instead of EMR-based data.

The accuracy of our EMR race and ethnicity data is similar to the accuracy found in other reports in the literature (88%) (6–17), although our analysis included a diverse, majority–minority population and data that were collected over a longer period. The accuracy of racial and ethnic classification in our hospital’s EMR system for non-White populations (86%) was greater than the accuracy reported in the literature (66% for non-White) (6–17).

Several factors may explain the discordance between our survey results and hospital EMR-based racial and ethnic categories. First, despite standardized hospital procedures to identify race and ethnicity, inconsistencies in data collection may exist. These inconsistencies may apply particularly to patients who are critically ill, cannot speak English, or have difficulty communicating (6–17). Second, the assumption that the race with which a person most identifies is fixed over time, and thus does not need to be confirmed at subsequent visits, may not be appropriate. However, this discrepancy does not account for the 5.1% (43 of 847) of patients who indicated an EMR-based race or ethnicity that was not among any of the self-identified races or ethnicities reported during the survey. Third, it may not be reasonable to expect a single race and ethnicity to fully describe a person’s identity, especially a person who is multiracial. The US Department of Health and Human Services acknowledges that the most recent data standards published in 2011 might not work in “other contexts,” such as administrative records that allow for a single entry only (4). This shortcoming is especially important for groups such as Native Hawaiians, who have access to resources (such as special programs and funding) devoted to any person who has Native Hawaiian lineage. Thus, estimates involving Native Hawaiian people often intend to capture a broader audience than estimates comprising people who identify as Native Hawaiian only. Other strategies, such as allowing for a separate indicator variable for populations of interest, may be necessary to ensure a complete census and appropriate allocation of resources.

Our results address a major gap in the literature by determining the accuracy of EMR-based data on race and ethnicity in a highly diverse population, including people who are most likely to experience health inequities, and they demonstrate the potential impact of misclassification of race and ethnicity in health research. Our findings have broad implications for public health. First, with 86.5% congruence between EMR-based and self-identified race and ethnicity, our findings suggest that EMR-based data are generally accurate. However, the lower accuracy among multiracial patients than among nonmultiracial patients highlights the need to reinforce or modify the standardized approach to collection of data on race and ethnicity. Second, the number of people who self-identify as multiracial is rapidly growing, and this population is becoming increasingly diverse. Although our results suggest that the accuracy of EMR-based data may be lower among multiracial populations than among nonmultiracial populations, it is not clear what the correct approach should be for collecting data on multiracial populations. What is the preferred approach if multiple categories of race and ethnicity are allowed in the collection of data for public health purposes? What if a multiracial person does not want to choose a single race or ethnicity? We emphasize that accuracy is diminished not because people are multiracial but because systems are not set up to capture data on race and ethnicity for this population.

### Limitations

Our study is subject to several limitations. First, our projections were based on self-identification of race and ethnicity in a sample of patients seeking care in a single hospital with a strong commitment to the health of Native Hawaiians and Pacific Islanders, and our results may be less generalizable to other hospitals within and outside Hawaii. However, QMC is the largest hospital in the state and accounted for 45% of all COVID-19–related hospitalizations. Moreover, the demographic characteristics of COVID-19 patients at QMC were similar to the demographics of the population in Hawaii. Second, we aggregated race and ethnicity categories to broader categories to explore the impact of our findings on state-reported data. This process resulted in the loss of specificity and, thus, may weaken our claim that our reference group was the gold standard. Third, it is possible that our projections would be less accurate for other COVID-19–related indicators, such as COVID-19 vaccinations, cases, and deaths, where race- and ethnicity-stratified data may be collected in a variety of ways rather than solely through the hospital’s EMR system. Fourth, there may be confounders in our self-identification projections. Multiracial people are more likely to be younger than 18 years, but younger people are also less likely to be hospitalized with COVID-19. We demonstrated that age did not affect the misclassification rate. However, our data set did not permit us to assess the confounding potential of other risk factors of COVID-19 hospitalizations such as comorbidities, occupation, or likelihood of vaccination. Individual-level COVID-19 hospitalization data with more covariates may have led to more accurate projections. Finally, all single-category racial and ethnic classification systems assume that racial identity is a static and singular entity and make no distinctions among the wide range of experiences, backgrounds, and needs of individuals.

### Conclusion

In a multicultural, majority–minority population of patients in a hospital in Hawaii, the accuracy of race and ethnicity in the hospital EMR system was 86.5% when compared to the gold-standard of self-reported race and ethnicity. Multiracial patients were significantly more likely than nonmultiracial patients to be miscategorized. When we projected this misclassification onto state-level COVID-19 hospitalization data in Hawaii, we found larger health disparities by race and ethnicity among Native Hawaiians and Pacific Islanders. Thus, race and ethnicity misclassification in hospital EMR records may mask the true burden of disease among Native Hawaiians and Pacific Islanders. Further research is needed to determine whether these findings are generalizable to other racial and ethnic groups in other geographical areas.
